# Longitudinal melanonychia in childhood: a great challenge^⋆^

**DOI:** 10.1016/j.abd.2021.02.012

**Published:** 2022-06-09

**Authors:** Isabela Boechat Morato, João Renato Vianna Gontijo, Glaysson Tassara Tavares, Flávia Vasques Bittencourt

**Affiliations:** a Hospital das Clínicas, Universidade Federal de Minas Gerais, Belo Horizonte, MG, Brazil; b Faculty of Medicine, Universidade Federal de Minas Gerais, Belo Horizonte, MG, Brazil

Dear Editor,

Longitudinal melanonychia (LM) is a longitudinal pigmentation of the nail plate. It occurs due to the increased production of melanin in the nail matrix, either by activation or proliferation of melanocytes. It is more prevalent after the fifth decade of life and uncommon in childhood.^1^ When it results from the activation of melanocytes, it takes on a grayish color, and its main etiologies are ethnic pigmentation, trauma, onychotillomania, medication, and genetic syndromes, such as Peutz-Jeghers and Lauzier-Hunziker. When secondary to melanocyte proliferation, it shows a brownish- black color and it is due to melanocytic nevus (MN), lentigo simplex, atypical melanocytic hyperplasia, or melanoma.^2^

While approximately 5% of LM in adults correspond to melanoma, it is typically benign in childhood, with MN being one of the major causes, but it shares clinical, dermoscopic, and histopathological features with subungual melanoma.^3^, ^4^ Nail matrix biopsy is the gold standard for etiological diagnosis, but it is an invasive procedure, difficult to perform in children, often complicated by nail dystrophy and recurrence, which makes the decision between clinical follow-up and biopsy to be challenging.

Hutchinson's sign corresponds to periungual pigmentation (a classic risk sign for subungual melanoma in adults), and micro-Hutchinson's sign is periungual pigmentation seen only at dermoscopy, both of which are commonly found in childhood LM. The pseudo-Hutchinson sign corresponds to the pigmentation of the nail matrix visible through the cuticle, thus not being a true warning sign.^2^, ^5^

A series of four cases of LM in children are described, highlighting the peculiarities of this entity in childhood.

## Case 1

**Figure 1:**
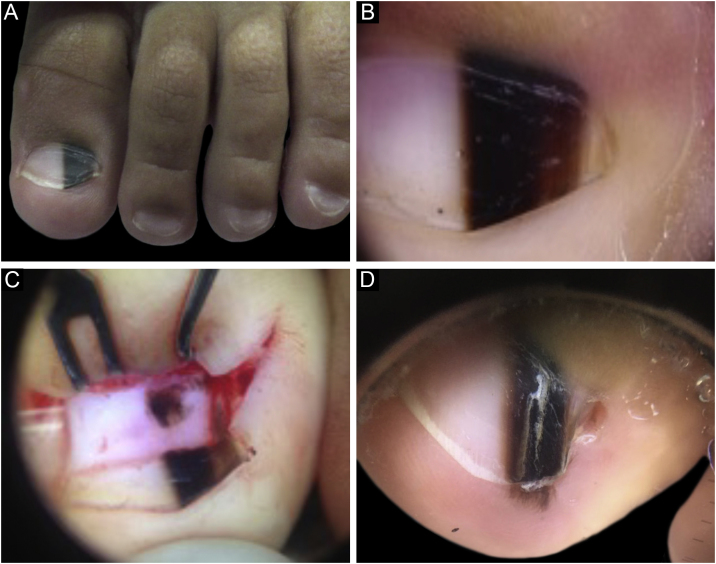
(A), Clinical aspect of the LM, taking up 50% of the total thickness of the left hallux nail. (B), Dermoscopy at the first consultation. (C), Intraoperative dermoscopy, showing a pigmented lesion with an irregular pattern and eccentric blur. (D), Dermoscopy two years after the biopsy, Hutchinson's sign in the hyponychium.

A two-year-old male patient, presented with a single LM on the left hallux that started at six months of age, with progressive growth. A light and dark brown band occupied 40% of the nail plate width, showing a triangular shape. Dermoscopy disclosed irregular lines regarding color, thickness and spacing, with blurred lateral edges. Intraoperative dermoscopy showed an irregular pattern. An excisional biopsy was performed, and histopathology was compatible with a junctional lentiginous ungual MN. The patient developed nail dystrophy after the biopsy and the melanonychia recurred two years later, with the presence of Hutchinson's sign in the hyponychium. Patient follow-up was chosen, with stability throughout the four-year follow-up (Fig. 1).

## Case 2

**Figure 2:**
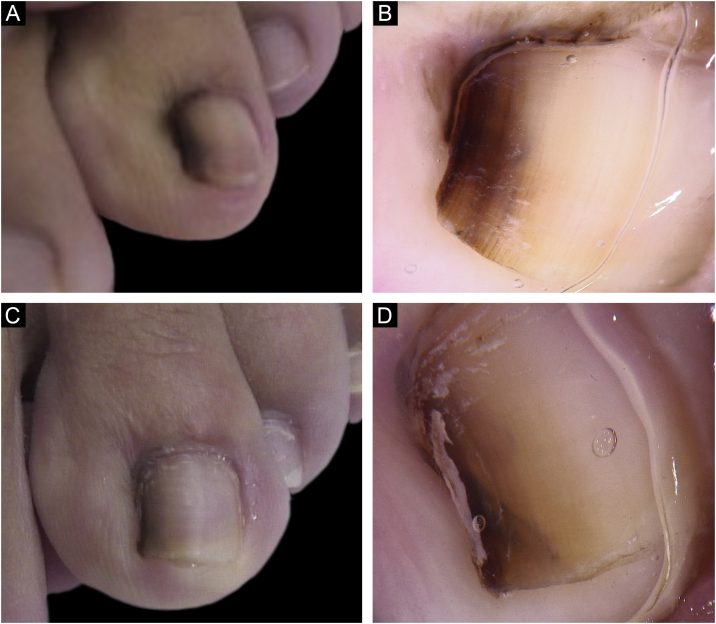
Clinical (A) and dermoscopic (B) aspect at the first consultation; (C–D) Clinical (C) and dermoscopic (D) aspect five years after the biopsy.

A 13-year-old female patient with a single LM present at birth and showing progressive growth. There was a light and dark brown band on the second left toe, measuring 3 mm, which occupied 20% of the nail width, with the presence of Hutchinson's sign in the proximal and lateral nail folds. Dermoscopy showed irregular lines in thickness and color, with loss of parallelism and blurred edges. A tangential biopsy was performed, which was compatible with a junctional MN. After the biopsy, the LM persisted on the lateral nail border without dystrophy and remained stable throughout five years of follow-up (Fig. 2).

## Case 3

**Figure 3:**
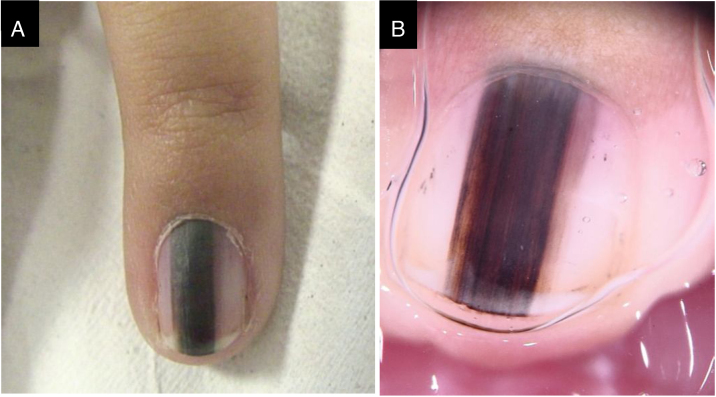
(A), Clinical aspect. (B), Dermoscopy: irregular color and thickness, loss of parallelism and blurred edges.

A three-year-old female patient with LM on the fifth finger of the left hand for two years, with recurrence after a previous biopsy performed one year had shown a lesion compatible with a junctional MN on histopathology, and which had grown since then. A black to dark brown band occupied 50% of the nail width, with pseudo-Hutchinson's sign. Dermoscopy showed irregular lines regarding color and thickness, loss of parallelism, and blurred edges. A second evaluation of the histopathological examination did not detect any signs of malignancy. The LM remained stable throughout two years of follow-up (Fig. 3).

## Case 4

**Figure 4:**
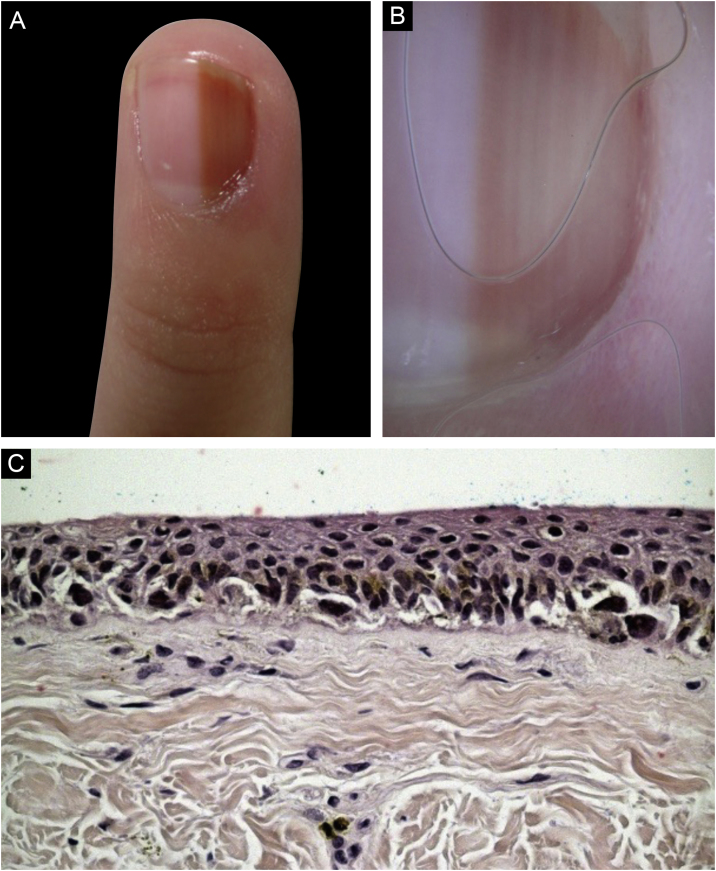
(A-B), Clinical (A) and dermoscopic (B) aspect at the first consultation; (C), histopathology examination stained with H&E showing melanocyte proliferation in a continuous arrangement forming some nests at the dermoepidermal junction.

A nine-year-old female patient, presenting a LM for six years on the third left finger, with progressive growth. A light brown band was observed, which took up 40% of the nail plate (4 mm), with pseudo-Hutchinson's sign. Dermoscopy showed homogeneous lines, preserving parallelism. Follow-up was chosen, but there was a loss of follow-up, and the patient returned four years later, with a biopsy performed in another service compatible with acral lentiginous melanoma *in situ*. A second evaluation of the histopathology favored the hypothesis of lentiginous MN, but it was not possible to rule out melanoma *in situ*. The margins were widened by 5 mm, and the ungual complex was removed up to the periosteal level, with healing by second intention. She was maintained under strict clinical follow-up, with no signs of recurrence or metastasis during eight years of follow-up.(Fig. 4)

In adults, clinical warning signs for subungual melanoma are considered to be the recent appearance of LM in a single nail, with heterogeneous coloration, history of changes in color, thickness or shape, width greater than 2/3 of the nail or greater than 3 mm, proximal width greater than the distal one (triangular shape), Hutchinson's sign, and associated nail dystrophy. Warning dermoscopic findings include heterogeneous brown to black color, irregular lines (regarding color, spacing, thickness, and loss of parallelism), blurring of edges, and micro-Hutchinson's sign.^2^, ^6^

However, such findings are common in childhood LM, without translating into an increased risk of malignancy. The MN, a major cause of LM in children, often presents with rapid growth, wide bands, heterogeneous pigmentation, irregular lines, triangular shape, and Hutchinson's sign, as demonstrated in other studies.^3^, ^4^, ^7^, ^8^, ^9^, ^10^ Its recurrence after excision is frequent, as in Cases 1 and 3. Subungual melanoma is extremely rare in children, with few reports in the literature, all *in situ*. Interestingly, Case 4, reported as subungual melanoma *in situ*, had less clinical and dermoscopic suspicion during the initial evaluation. The biopsy showed lentiginous melanocytic proliferation with rare nests, focal cytologic atypia, and preserved symmetry. Acral MN may present with cytoarchitectural atypia without indicating malignancy, while subungual melanoma may have few atypia in the initial phase, which makes the differential diagnosis between these two entities a difficult one. Considering the preservation of histological symmetry and the long period of 10 years of evolution with no evidence of progression to invasive melanoma, Case 4 is more likely to represent an MN than an *in situ* melanoma.

The cases described herein illustrate the challenge in managing children with LM. Although the appearance and behavior of the lesion can be challenging, simulating melanoma, some authors disagree with the systematic indication of biopsy.^9^ Management should be individualized, prioritizing clinical and dermoscopic follow-up, in addition to considering the family's level of anxiety. When, however, the biopsy is chosen, it is important that it is guided by intraoperative dermoscopy and performed tangentially when possible, minimizing the risk of nail distrophy. When facing an irregular pattern on intraoperative dermoscopy, however, excisional biopsy may be chosen, aiming at a lower risk of recurrence. Moreover, the analysis by a pathologist with expertise in melanocytic lesions is imperative.

## Financial support

None declared.

## Authors' contributions

Isabela Boechat Morato: Design and planning of the study, data collection, drafting and editing of the manuscript, critical review of intellectual content, approval of the final version of the manuscript.

João Renato Vianna Gontijo: Drafting and editing of the manuscript and critical review of the relevant intellectual content, approval of the final version of the manuscript.

Glaysson Tassara Tavares: Drafting and editing of the manuscript and critical review of the relevant intellectual content, approval of the final version of the manuscript.

Flávia Vasques Bittencourt: Drafting and editing of the manuscript and critical review of the relevant intellectual content, approval of the final version of the manuscript.

## Conflicts of interest

None declared.
